# Patterns and determinants of modern contraceptive use and intention to usecontraceptives among Malawian women of reproductive ages (15–49 years)

**DOI:** 10.1186/s40834-021-00163-8

**Published:** 2021-07-01

**Authors:** James Forty, Serai Daniel Rakgoasi, Mpho Keetile

**Affiliations:** Department of Population Studies, University of Botswana, Private Bag UB, 00705 Gaborone, Botswana

**Keywords:** Determinants, Modern contraceptive use, Malawi, Women, Reproductive age

## Abstract

**Background:**

Malawi is one of the countries in SSA with the highest TFR. This study aimed to explore factors associated with modern contraceptive use and intention to use contraceptives among women of reproductive ages (15–49 years) in Malawi.

**Methods:**

The study used secondary data from 2015 to 16 Malawi Demographic and Health Survey (MDHS) dataset. Logistic regression models were used to derive adjusted odd ratios as the measures of association between need, predisposing and enabling factors, and contraceptive use and the intention to use contraceptives among women. The sample constituted 24,562 women who were successfully interviewed during the MDHS. All comparisons are considered statistically significant at 5% level.

**Results:**

Overall 54.8% of women were currently using contraceptives, while 69.1% had the intention to use contraceptives. The odds of contraceptive use were significantly low among, women aged 15–19 years, 20–24 years, 25–29 years, 30–34 years, 35–39 years and 40–44 years compared to women aged 45–49 years; women of Tonga ethnic group (OR = O.60, CI = 0.43 0.84) compared to women of Nyanga ethnic group; women from poor households (OR = 0.78, CI = 0.68–0.90) and middle income households (OR = 0.84, CI = 0.74–0.95) compared to women from rich household. Nonetheless, women with no past experience of terminated pregnancy (OR = 1.50, CI = 1.34–1.68) were more likely to use contraceptives compared to women with past experience of terminated pregnancy. Similarly, Women with primary education (OR = 1.56, CI = 1.16–2.09) and secondary education (OR = 1.39, CI = 1.04–1.85) were more likely to use contraceptives compared to women with higher education. While the odds of intending to use contraceptives were significantly high with age only thus among women aged 15–19 years, (OR = 15.18, CI = 5.94–38.77); 20–24 years (OR = 16.77, CI = 7.46–37.71); 25–29 years (OR = 6.75, CI = 3.16–14.45); 30–34 years (OR = 7.75, CI = 3.61–16.65) and 35–39 years (OR = 5.05, CI = 2.29–11.12) compared to women aged 45–49 years.

**Conclusion:**

As direct policy measure; information, education and communication programmes on family planning among poor and middle income women, and all women in reproductive ages should be strengthened.

## Introduction

Family planning has improved the well-being of families and communities by preventing high risk pregnancy, abortion, and unplanned pregnancy and has the potential to reduce poverty and hunger as well as preventing maternal deaths and childhood deaths worldwide [1]. The main intervention in family planning is contraceptive use. The prevalence rates of contraceptive use tend to be higher in developed countries than in developing countries [2]. Among developing countries, the rates of contraceptive use vary widely and Sub-Saharan Africa (SSA) has the lowest prevalence rate of contraceptive use comparatively [3]. Lack of access to family planning services and concerns about the side effects associated with modern contraceptive methods are among the main reasons for the high unmet need of contraception in developing countries [4].

There are international commitments aiming at meeting the need to use contraceptives among women. Furthermore, there are important factors that help policy commitments to be realized. Studies done in developing nations, specifically in SSA found that contraceptive use and inte-ntion to use contraceptives were significantly associated with education, age, place of residence, religion, ethnicity, wealth index and husband’s or partner’s education [5–13]. Furthermore, women ever oriented on family planning by family planning workers and with past abortion experiences were also found to use contraceptive or with the intention to use contraceptives [8, 14, 15].

Malawi is committed to ensuring that contraceptive need and reproductive health needs in general are met. The country launched its first population policy in 1994. The current revised policy, now in its seventh year of implementation, was launched in 2013 and aims at addressing development challenges that emanate from unmanaged population growth and high levels of fertility and mortality [16]. According to Population policy and Family planning commitment (2020) there is an increase in contraceptive use. Thus, Malawi’s contraceptive use prevalence rate increased from 7% in 1992 to 28% in 2004 and had reached 58% in 2015–16. Thus, by 2016, Malawi had nearly managed to attain 60% modern contraceptive prevalence rate as targeted by Family Planning Commitment (2020). Further, with estimated total fertility rate (TFR) of 4.2 in 2016 according to the Malawi Demographic Health Survey (MDHS,2015–16), Malawi was at the verge of attaining the target of National Sexual and Reproductive Health Strategy, 2011–2016 of 4.0 TFR by 2016.

Despite the progress, Malawi is one of the countries in SSA with the highest TFR but at the same time with higher contraceptive prevalence rate (CPR). For example, Malawi’s TFR is 4.2 compared to South Africa’s (2.6), Kenya’s (3.9) and Gabon’s (4.1) yet Malawi’s CPR (58%) is similar to Kenya’s and higher than those of the other nations [17]. This shows that Malawi has slow decline in TFR hence population continues to grow rapidly. It is against this background that this paper intends to establish factors that may inhibit realization of desired low fertility which can mainly be attained through contraceptive use. The realization of the factors that are distorting contraceptive use and its translation to desired fertility may help to come up with necessary interventions to deal with the same. Therefore, this study aims at finding determinants of modern contraceptives use and intention to use contraceptives.

Studies in Malawi found out contraceptive use is significantly associated with age, education, spouse approval by, number of living children, work status, visiting health facility and discussionbetween spouse and respondent, region, place of residence, parity, marital status and religion [18–21]. However, most studies, including Mandiwa et al. [19] focused on adolescents only. Meanwhile Palamuleni [20] focused on all women of reproductive ages, but the study used a 2010 MDHS dataset. On the other hand, Makupe et al. [18] focused on all women of reproductive age as well used the dataset that this study has used but there are differences in methods and in some explanatory variables used. For instance on finding the determinants, Makupe [19] used mixed effect model while this study used logistic regression. Furthermore, the two studies also differ in explanatory variables except on age, religion, region, place of residence and respondent education. Nonetheless, these common variables may be important for triangulating the results where two different method or models were used. Hence, this study is important in triangulating the findings of other studies and further finding other determinants that contribute to the prevailing high fertility irrespective of relatively high contraceptive prevalence rate in Malawi. Additionally, studies are scarce in Malawi pertaining intention to use contraceptives. Thus, the second aim of this study, as already alluded to, is to establish if the same explanatory variables under consideration are associated with intention to use contraceptives.

## Conceptual framework

In order to assess the patterns and determinants of modern contraceptive use among women of reproductive ages in Malawi, this study adopted the theory of health-seeking behavior developed by Andersen and Newman [22]. The key focus was to examine the relationship between intra and inter-personal factors and contraceptive use. The main premise of the theory of health seeking behavior is that the use of health-care services is a function of three sets of individual characteristics; predisposing, enabling, and need factors. Predisposing factors are considered to be the individual and socio-cultural characteristics such as social structure, health beliefs and demographic factors [22]. Enabling factors for contraceptive use may include personal, family, place of residence and community resources which may increase accessibility to family planning resources. For instance; income, employment and the availability of family planning services could be categorized as enabling factors [22]. Need factors are self-perceptions and objective evaluations of general health conditions [23]. Several studies have suggested that abortion expe-rience and desire to have additional children are two crucial need factors related to contraceptive use [15, 24].

**Fig. 1:**
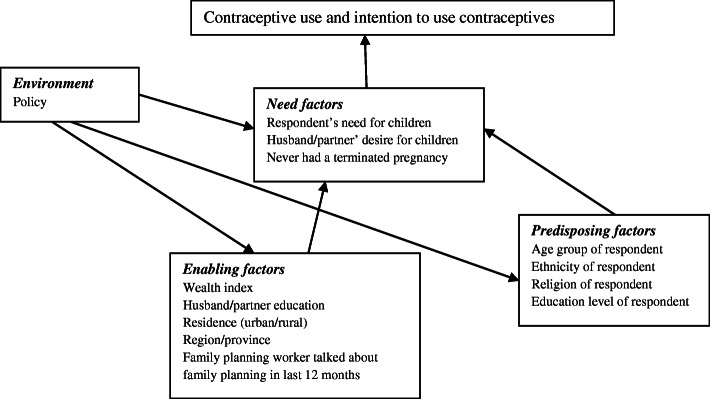
Interaction of predisposing factors, enabling factors and needs factors and contraceptive use and intention to use

Figure 1 shows the pathways through which predisposing, enabling and need factors influence contraceptive use and intention to use contraceptives among women of reproductive age. For example the enabling factors like good knowledge of fertility awareness, household of higher wealth index, partners with higher education and living in urban [25, 26] can have some influence on the need of having few numbers of children which in turn affect contraceptive use. On the other hand, predisposing factors like education of women, age, religion and ethnicity tend to influence the decision on how many children a woman wants to have, which in turn affects contraceptive use [27–29]. Moreover, Malawi’s 2013 population policy creates an enabling environment for increase in contraceptive use and intention to use contraceptives. For instance, the second specific objective of policy area 1 is the promotion of benefits of having few children [26]. The attainment of having few children is through the third and fourth specific objectives of policy area 1 which is ensuring reproductive health information and interventions are given and accessible to all irrespective of predisposing factors (age, ethnicity, religion and education) or enabling factors (wealth index, place of residence and partner education). Thus, such a policy has an overarching effect as it influences the need to have less number of children on both men and women irrespective of variations in predisposing and enabling factor.

## Methods

### The study area

Malawi is a landlocked, long stretched country in southeastern Africa in the Great Rift Valley on the western shore of Lake Nyasa (Lake Malawi), the most southerly lake in the Great African Rift Valley system. Countries which borders with Malawi are Tanzania, Zambia and Mozambi-que. Malawi is one of the world’s most densely populated nations with a population of about 17.6 million people according to 2018 Census report. Capital city is Lilongwe and English and Chichewa are the official languages. The nation is divided into three main regions: Northern, Central and Southern. Malawi’s population is predominantly rural and is comprised of various ethnic groups that migrated from other parts of Africa. Based on its gross national income (GNI) the Republic of Malawi is one of the poorest country in Africa, its economy is agro-based. There is a small tourism sector which is being primed for growth. The former British protectorate of Nyasaland became the independent nation of Malawi in 1964. First democratic multiparty elections were held in 1994, after three decades of one-party rule.

### Study design and data source

This study used secondary data cross sectional household data for women collected during the 2015–16 Malawi Demographic and Health Survey (MDHS). The data used in this analysis were collected using the women’s individual questionnaire. The MDHS is designed to provide data for monitoring the population and health situation in Malawi [30].

The sampling frame used for the 2015–16 MDHS was derived from the Malawi Population and Housing Census frame (MPHC), conducted in Malawi in 2008, and provided by the Malawi National Statistical Office (NSO). The census frame is a complete list of all census standard enumeration areas (SEAs). SEA is a geographic area that covers an average of 235 households. The sampling frame contains information about the SEA location, type of residence (urban or rural), and the estimated number of residential households. The 2015–16 MDHS sample was stratified and selected in two stages. Each district was stratified into urban and rural areas; this yielded 56 sampling strata. Samples of SEAs were selected independently in each stratum in two stages. In the first stage, 850 SEAs, including 173 SEAs in urban areas and 677 in rural areas, were selected with probability proportional to the SEA size and with independent selection in each sampling stratum. A household listing operation was implemented in all the selected SEAs and the lists of households served as a sampling frame for the selection of households in the second stage.

In the second stage of selection, a fixed number of 30 households per urban enumeration area and 33 per rural enumeration area were selected with an equal probability systematic selection from the newly created household listing. All women aged 15–49 years who were either perma-nent residents of the selected households or visitorswho stayed in the household the night before the survey were eligible to be interviewed. Thus, in the 850 selected clusters, 26,564 households were occupied at the time of data collection of which 26,361 were successfully interviewed, yielding a household response rate of 99%. In total, 24,562 women were successfully interviewed andincluded in this analysis.

### Study variables and measurements

#### Dependent variables

The outcome variables of this study are contraceptive use and intention to use contraceptives derived from questions which asked women “do you or your partner use contraceptives or intend to use contraceptives to delay or avoid getting pregnant”. For contraceptive use, women who reported current use of either modern contraceptive method were given a code of **1** and **0** if otherwise. For the intention to use contraceptives, women who intended to use contraceptive were given a code of **1** and those who responded did not intend to use were coded **0**.

#### Independent variables

The independent variables were grouped into three categories; predisposing, enabling and need variables. Predisposing factors are individual and socio-cultural characteristics such as social structure, health beliefs and demographic factors [22]. Predisposing variables included age group (in categories of; 15–19 and 20–24, 25–29, 30–34, 35–39, 40–44 and 45–49), ethnicity (Mang’anjaChewa, Tubuka, Lomwe, Tonga, Yao, Sena, Nkhonde, Ngoni, and Nyanga), religion (Christians, Muslims, no religion) and education level (no education, primary, secondary and higher). This was done consistent with previous literature [9–12, 20, 31].

Enabling factors reflect the means or logistics required to obtain or increase accessibility to the services [22]. Enabling variables included wealth index (poor, rich and richest), husband/partner level of education (no education, primary, secondary and higher), residence (urban and rural), family planning worker visitation and having talked on family planning in last 12 months and region (northern, central and south). These variables were selected on the basis of reviewed literature [5–8, 10, 12, 31].

Need factors are self-perception of desired condition [23]. Need factors selected include desire for children (want the same, husband/partner wants more and husband or partner wants fewer) and experience of terminated pregnancy. Studies show past abortion experiences [24] and respondent or partner’s desire for children are associated with contraceptive use [15].

### Data analysis

Prior to data analysis, the data was cleaned and some variables; contraceptive use and intention to use, religion and wealth index were re-coded to suit the objectives of this study. Contraceptive use and the intention to use contraceptives were analyzed separately. Descriptive statistics was used to come up with summarized univariate data and the results were presented as proportions (%). Bivariate analysis was done where enabling, predisposing and need factors were crosstabu-lated with contraceptive use and the intension to use contraceptive. Pearson Chi-Square was usedto determine the significant explanatory factors for regression analysis. Binary logistic regression analysis was employed to identify predictors of contraceptive use and intention to use. Crude andadjusted odds ratios and their 95% confidence intervals (95% CI) were estimated. All statistical analyses were performed using Statistical Package for the Social Sciences (SPSS, IBM version 25) and statistical significance was set at *P*-value of less than 0.05.

## Results

### Sample description

Table 1 gives the summary statistics of the study population. The results indicate that 54.8% (11,194/24562) of women in the sample reported that they used contraceptives. Among those who indicated that they did not use any contraceptives, 69.1% (4126/9242) expressed the intention to use contraceptives. Age distribution of respondents shows that, the highest proportion of women in the sample was found in ages 15–19 and 20–24 years (21.5 and 20.7%, respectively). It was also noted that the Chewa ethnic group (30.8%), Christians (88.4%), primary education level (61.2%), middle income individuals (38.3%) and rural residents (78.6%) constituted the highest proportions in the sample. Moreover, the proportion of women whose partner had primary education (53.2%), were educated on family planning (90%), had the desire to have the same number of children as their partner (69.7%), and those who never terminated pregnancy (90%) were high in the sample.

**Table 1:** Distribution of sampled women by socioeconomic and demographic characteristics, Malawi DHS, 2015/16

Variable	N	%
**Predisposing factors**		
***Age***		
15–19	5273	21.5
20–24	5094	20.7
25–29	3976	16.2
30–34	3648	14.9
35–39	2988	12.2
40–44	2022	8.2
45–49	1561	6.4
***Ethnicity***		
Chewa	7317	30.8
Tumbuka	2612	11.0
Lomwe	4453	18.7
Tonga	942	4.0
Yao	2782	11.7
Sena	1153	4.8
Nkhonde	335	1.4
Ngoni	3085	13.0
Mang’anja	569	2.4
Nyanja	547	2.3
***Religion***		
Christian	21,685	88.4
Moslem	2726	11.1
Non-religious	123	0.5
***Education level***		
No education	2779	11.3
Primary	15,028	61.2
Secondary	6061	24.7
Tertiary	694	2.8
**Enabling factors**		
***Wealth status***		
Poor	8708	35.5
Middle income	9405	38.3
Rich	6449	26.3
***Partner education level***		
No education	1456	9.2
Primary	8377	53.2
Secondary	5008	31.8
Tertiary	915	5.8
***Place of residence***		
Urban	5247	21.4
Rural	19,315	78.6
***Educated on family planning***		
No	325	10.0
Yes	2926	90.0
***Region***		
Northern region	4803	19.6
Central region	8417	34.3
Southern region	11,342	46.2
**Need factors**		
***Desire of children***		
Both want same	8605	69.7
Partner wants more	2540	20.6
Partner wants fewer	1206	9.7
***Ever terminated pregnancy***		
No	22,102	90.0
Yes	2460	10.0
**Outcome variables**		
***Contraceptive use***		
No	9242	45.2
Yes	11,194	54.8
***Intention to use contraceptive***		
No	4126	30.1
Yes	9242	69.9
Total	24,562	100

### Patterns of contraceptive use and intention to use

#### Contraceptive use

Contraceptive use was significantly associated with age group, ethnicity, religion, women education level, wealth index, husbands/partners’ education level, type of place of residence (urban/rural), region and experience of terminated pregnancy (Table 2). For example, contrace-ptive use increased with age and was highest in the age group 45–49 years (81.1%). Furthermore, a high proportion (61.0%) of women who belonged to Mang’anja ethnic group, non-religious (58.0%), not educated (65.1%) and belonged to middle income households (55.9%) used contraceptive. Moreover, women whose partners had tertiary education (71.8%), resided in rural areas (55.4%), or resided in northern region (56.8%) and experienced terminated pregnancy (61.8%) used contraceptives.

**Table 2:** Prevalence of contraceptive use and intention to use contraceptives by predisposing, enabling and need factors, 2015–16 MDHS (*N* = 24,562)

Variable	***Contraceptive use***		***Intension to use contraceptives***	
	N	%	N	%
**Predisposing factors**				
***Age***				
15–19	810	19.2	3402	76.2
20–24	2377	51.0	2286	84.1
25–29	2295	63.7	1309	77.9
30–34	2136	66.7	1065	70.4
35–39	1807	71.1	698	59.1
40–44	1091	76.7	331	35.6
45–49	678	81.1	151	17.1
*P-value*	*< 0.001*		*< 0.001*	
***Ethnicity***				
Chewa	3499	55.5	2809	73.6
Tumbuka	1167	56.6	895	61.9
Lomwe	2176	56.8	1655	72.7
Tonga	345	47.1	387	64.8
Yao	1049	48.3	1125	64.9
Sena	495	52.3	452	68.7
Nkhonde	150	54.2	127	68.6
Ngoni	1411	54.8	1166	69.7
Mang’anja	288	61.0	184	65.5
Nyanja	226	52.8	202	62.9
*P-value*	*< 0.001*		*< 0.001*	
***Religion***				
Christian	10,107	55.6	8079	69.8
Moslem	1020	47.8	1112	65.2
Non-religious	51	58.0	37	59.7
P-value	< *0.001*		*< 0.001*	
***Highest education level***				
No education	1319	65.1	708	48.5
Primary	7148	56.7	5468	69.4
Secondary	2456	47.0	2774	76.9
Tertiary	271	48.1	292	69.0
*P-value*	*< 0.001*		*< 0.001*	
**Enabling factors**				
***Wealth group***				
Poor	3884	54.9	3287	69.7
Middle income	4408	55.9	3471	69.5
Rich	2792	52.9	2484	67.9
*P-value*	*0.003*		*0.170*	
***Partner education level***				
No education	784	67.4	379	56.4
Primary	4999	68.4	2309	68.4
Secondary	2987	66.0	1542	76.3
Tertiary	574	71.8	225	66.0
*P-value*	*< 0.001*		*< 0.001*	
***Type of place of residence***				
Urban	2282	52.4	2077	70.1
Rural	8912	55.4	7165	68.9
*P-value*	*< 0.001*		*0.221*	
***Educated on family planning***				
No	144	56.7	110	60.8
Yes	1602	62.2	975	73.6
*P-value*	*0.087*		*< 0.001*	
***Region***				
Northern region	2168	56.8	1649	62.6
Central region	3969	54.1	3368	75.7
Southern region	5057	54.5	4225	62.7
*P-value*	*0.018*		*< 0.001*	
**Need factors**				
***Desire of children***				
Both want same	4839	64.3	2681	71.2
Partner wants more	1403	64.9	758	66.7
Partner wants fewer	727	66.2	371	77.5
*P-value*	*0.463*		*< 0.001*	
***Ever terminated pregnancy***				
No	9967	54.1	8456	69.7
Yes	1227	61.8	786	63.7
*P-value*	*< 0.001*		*< 0.001*	

Statistically significant at P < 0.05

#### Intention to use contraceptives

Intention to use contraceptives was significantly associated with age group, ethnicity, religion, women’s education level, husbands/partners’ education level, educated on family planning, region, desire for children and experience of terminated pregnancy (Table 2). For example, intention to use contraceptives decreased with age and was highest in age group 20–24 (84.1%) and lowest in age group 45–49 (17.1%%). Furthermore, a high proportion of women who belonged to Chewa ethnic group (73.6%), Christians (69.8%), poor households (69.7%) and secondary education (76.9%) intended to use contraceptive. Meanwhile, women whose partners had tertiary education (73.6%), resided in the Central region (75.7%), partner wanted fewer number of children (77.5%) and never experienced terminated pregnancy (69.7%) intended to use contraceptives.

### Determinants of contraceptive use and intention to use

Table 3 shows the odds ratios for the likelihood of contraceptive use and intention to use contraceptives among women in Malawi.

**Table 3:** Adjusted odds ratios showing the likelihood of contraceptive use and intention to use among women (15–49 years) in Malawi

Variable	Contraceptive use				Intention to use contraceptives			
	Unadjusted Model		Adjusted Model		Unadjusted Model		Adjusted model	
	OR	95% C.I.	OR	95% C.I.	OR	95% C.I.	OR	95% C.I.
***Age***								
15–19	0.05***	0.04–0.06	0.13***	0.10–0.16	15.54***	12 .88–18.76	15.18***	5.94–38.77
20–24	0.23***	0.19–0.28	0.24***	0.19–0.31	25.71***	20.98–31.50	16.77***	7.46–37.71
25–29	0.39***	0.32–0.47	0.37***	0.29–0.46	17.06***	13.83–21.04	6.75***	3.16–14.45
30–34	0.45***	0.37–0.54	0.40***	0.32–0.51	11.55***	9.39–14.21	7.76***	3.61–16.65
35–39	0.58***	0.47–0.70	0.55***	0.43–0.70	7.01***	5.68–8.64	5.05***	2.29–11.12
40–44	0.73***	0.59–0.91	0.73***	0.56–0.94	2.67***	2.15–3.34	2.04	0.89–4.65
45–49	1.00		1.00		1.00		1.00	
***Ethnicity***								
Chewa	1.11	0.92–1.36	1.16	0.86–1.56	1.64***	1.29–2.08	0.97	0.20–4.15
Tumbuka	1.17	0.95–1.44	1.09	0.80–1.48	0.96	0.75–1.23	0.48	0.12–2.02
Lomwe	1.18	0.96–1.44	1.25	0.92–1.69	1.57***	1.23–2.00	0.83	0.18–3.87
Tonga	0.80	0.63–1.01	0.60***	0.43–0.84	1.09	0.82–1.44	0.46	0.10–2.10
Yao	0.83	0.68–1.03	1.01	0.73–1.40	1.09	0.85–1.40	0.43	0.08–2.18
Sena	0.98	0.78–1.23	0.87	0.63–1.21	1.29	0.98–1.71	0.65	0.11–3.84
Nkhonde	1.06	0.78–1.43	0.92	0.60–1.40	1.29	0.88–1.90	1.29	0.20–8.44
Ngoni	1.08	0.88–1.33	1.12	0.83–1.52	1.35***	1.05–1.74	0.80	0.17–3.67
Mang’anja	1.40***	1.07–1.83	1.43	0.97–2.10	1.12	0.88–1.56	0.52	0.06–4.61
Nyanja	1.00		1.00		1.00		1.00	
***Religion***								
Christian	0.91	0.59–1.39	1.07	0.62–1.86	1.56	0.94–2.60	1.02	0.61–1.82
Moslem	0.67	0.43–1.03	0.72	0.41–1.26	1.27	0.75–2.12	0.71	0.51–1.23
Non-religious	1.00		1.00		1.00		1.00	
***Highest education level***								
No education	2.01***	1.66–2.43	1.27	0.92–1.75	0.42***	0.34–0.53	0.75	0.16–3.65
Primary	1.41***	1.19–1.67	1.56***	1.14–2.09	1.02	0.82–1.26	0.72	0.16–3.18
Secondary	0.95	0.80–1.04	1.39***	1.04–1.85	1.50	1.20–1.87	0.77	0.18–3.34
Tertiary	1.00		1.00		1.00		1.00	
**Enabling factors**								
***Wealth group***								
Poor	1.08***	1.01–1.16	0.78***	0.68–0.90	0.98	0.81–1.16	0.74	0.68–1.35
Middle income	1.13***	1.05–1.21	0.84***	0.74–0.95	0.83	0.78–1.21	0.84	0.74–1.15
Rich	1.00		1.00		1.00		1.00	
***Partner education level***								
No education	0.81***	0.67–0.99	0.86	0.67–1.09	0.67***	0.51–0.87	1.08	0.34–3.38
Primary	0.85***	0.72–0.99	0.94	0.76–1.16	1.11	0.88–1.41	1.03	0.40–2.68
Secondary	0.76***	0.63–0.90	0.84	0.69–1.03	1.66***	1.30–2.12	1.14	0.18–2.92
Tertiary	1.000		1.000		1.00		1.000	
***Type of place of residence***								
Urban	0.88***	0.83–0.95	1.04	0.92–1.16	0.68	0.53–1.15	1.01	0.82–1.13
Rural	1.00		1.00		1.00		1.00	
***Region***								
Northern region	1.10***	1.02–1.19	1.09	0.91–1.30	0.82***	0.74–0.90	0.99	0.46–2.14
Central region	0.99	0.93–1.05	1.11	0.99–1.24	1.52***	1.40–1.66	0.66	0.36–1.23
Southern region	1.00		1.00		1.00		1.00	
**Need factors**								
***Ever terminated pregnancy***								
No	0.76***	0.69–0.83	1.50***	1.34–1.68	1.31	1.16–1.48	0.71	0.38–1.35
Yes	1.00		1.00		1.00		1.00	

***Statistically significant at *P* < 0.05

### Contraceptive use

After adjusting for covariates, women aged 15–19 years (OR = 0.13, CI = 0.10–0.16); 20–24 years (OR = 0.24, CI = 0.19–0.31); 25–29 years (OR = 0.37, CI = 0.29–0.46); 30–34 years (OR = 0.40, CI = 0.32–0.51), 35–39 years (OR = 0.55, CI = 0.43–0.70) and 40–44 years (OR = 0.73, *P* = 0.56–0.94) were less likely to use contraceptives compared to women aged 45–49 years. Furthermore, only women of Tonga ethnic group were less likely to use contraceptives (OR = O.60, CI = 0.43–0.84) compared to women of Nyanga ethnic group. Women with primary education (OR = 1.56, CI = 1.16–2.09) and secondary education (OR = 1.39, CI = 1.04–1.85) were more likely to use contraceptives compared to women with higher education. While women from poor households (OR = 0.78, CI = 0.68–0.90) and middle income households (OR = 0.84, CI = 0.74–0.95) were less likely to use contraceptives compared to women from rich households. On the other hand, women with no past experience of terminated pregnancy (OR = 1.50, CI = 1.34–1.68) were more likely to use contraceptives compared to women with past experience of terminated pregnancy.

### Intention to use

Most variables were not significantly associated with intention to use contraceptives except age. Women aged 15–19 years (OR = 15.18, CI = 5.94–38.77); 20–24 years (OR = 16.77, CI = 7.46–37.71); 25–29 years (OR = 6.75, CI = 3.16–14.45); 30–34 years (OR = 7.75, CI = 3.61–16.65) and 35–39 years (OR = 5.05, CI = 2.29–11.12) were more likely to intend to use contraceptives compared to women aged 45–49 years.

## Discussion

This study assessed the patterns and determinants of contraceptive use and intension to use contraceptives among women of reproductive ages in Malawi. The findings show that more than half of women reported that they were using contraceptives in 2015/16. This is an increase from the 2011 figure which was less than half (42%) [30]. The increase in contraceptive use observed in this study may have been contributed by, the USAID-funded Capacity-Plus Project implemented by IntraHealth International in collaboration with Christian Association of Malawi since January 2012 whose aim was to strengthen human resource management in affiliated health facilities in areas including contraceptives use [32]. Additionally, the inclusion of in-service training of health workers on long term family methods helped to reduce the shortage of health workers knowledgeable on contraceptives [32]. Thus, this may also have contributed to increase in CPR.

The results of the analyses show that contraceptive use increased with the age of women. For instance low contraceptive prevalence was observed among teenage women. The findings are consistent with previous findings which found that contraceptive use increased with age in Malawi [10, 20, 31, 33]. The low contraceptive prevalence among women in teen years may be due to the fact that most of these women especially aged below 18 may be shying away to seek contraceptives even if they are married as they fall below legal age (18 years) of marriage as well as not to be viewed as are indulging in premarital sex if they are not married.

On the other hand, intention to use contraceptives increased with age of women. This finding corroborates other findings in the region [20, 21]. The finding that women in teen years have higher intention to use contraceptives concurs with the already given explanation that they had low contraceptive use while they intended to use contraceptives. The reduction in intention to use contraceptives among older women may be related to the fact that a good number of older women might be not sexually active or they have reduced their coital frequency. Additionally, most of them may rely on other traditional methods like string tie and they may not be willing or comfortable to talk about them in an interview hence reported to have lower intention to use contraceptives.

Women of Tonga ethnic group were found to be less likely to use contraceptives. Studies also show that there is variation in contraceptives use among ethnic groups [11, 34]. The possible explanation for low contraceptives use among the Tonga ethnic group could be because the grouping is more likely to spend more time schooling hence tend to marry at late age thus need of children after marriage become a priority. The explanation is supported by Palamuleni [21] who found that Tonga ethnic group, despite being one of the smallest ethnic groups, the grouping is among the top 3 most educated ethnic groups and spend most time schooling hence most of them secure jobs and live in urban areas and marry late.

Contrary to previous findings [9, 10, 12, 31, 35] which found that increase in education relates to increase in contraceptive use; we found that women with low level of education were more likely to use contraceptives. The most plausible explanation for this observation is that in Malawi there is good coverage of reproductivehealth interventions among all women regardless of their education level. Furthermore, most women with higher education are likely to be married by educated men of their levels who tend to understand the subject of contraceptives hence they also tend to be proactive in using contraceptives like condoms hence giving option for their women for less utilization of contraceptives.

Women of poor and middle income wealth quintiles were less likely to use contraceptives. This could be because women with less than tertiary education hardly get employment especially well paying jobs hence often times they depend on their husbands/partners who dominate in decision making including contraceptive use. This is exacerbated by patriarchal system which obstructs women in decision making thus most women, except in most cases tertiary educated, are at the mercy of their partners. However, the finding is consistent with other studies [6, 7, 10, 12, 31], and]. For instance, Rasooly et al. found that women from wealthiest households were more likely to use contraceptives than women from less wealth households.

Women with no experience of terminated pregnancy were found to be more likely to use contra-ceptives. The finding is consistent with the finding by Khanal et al. [24] who found that women with no experience of terminated pregnancy were found to be more likely to use contraceptives. Nevertheless, the use of experience of past terminated pregnancy as a factor needs further debate as is tricky since it could either be intended artificial terminated pregnancy or unintended natural terminated pregnancy (miscarriages).

### Limitation of the study

Although our study provides vital insights on the association between predisposing, enabling and need factors and contraceptive use and on the intention to use contraceptives, it has some few limitations. For example, the outcome variables are explained based on women only yet most of them live with their husbands/ partners who most often are heads of households. As a result there is possibility of missing out on the male factor(s) that might have effect on contraceptive use and intention to use. Moreover, information was collected using self-reports, which can distort the accuracy of the results. Meanwhile, some variables on the influence of men on contraceptive use and intention to use contraceptives were included as need factors. For instance, partners desired number ofchildren. Despite these limitations, the study has shed some insights on the factors influencing contraceptive use and intention to use in Malawi among women aged 15–49 years*.*

## Conclusions

The findings of this study indicate that more than half of women in reproductive ages use contra-ceptives, while more than two thirds among those who were not using contraceptives expressed intention to use contraceptives. The odds of contraceptive use were significantly high among women in higher age groups, with primary and secondary education, from rich households and who had experience of terminated pregnancy. On the other hand, the odds of intention to use contraceptive were significantly high among women in lower age groups. The findings of this study have far-reaching policy implications that can improve the use of contraceptives. Such policies must include strategies to address impediments associated with women in lower ages with use of contraceptive. The policies should also include economic empowering strategies to households and women in particular to reduce socio-economic disparities among households. Additionally, there should be strategies targeting women with no formal education with family planning messages that include the use of contraceptives.

## Data Availability

The datasets during and/or analyzed during the current study is publicly available from the MEASURE DHS website.

## Abbreviations

CI: Confidence Interval
CPR: Contraceptive Prevalence Rate
DHS: Demographic and Health Survey
GNI: Gross National Income
MDHS: Malawi Demographic and Health Survey
MPHC: Malawi Population and Housing Commission
NSO: National Statistical office
OR: Odds Ratio
SEA: Standard Enumeration Area
SSA: Sub-Sahara Africa
TFR: Total Fertility Rate
